# Drying Kinetics and Chemical Properties of Mango

**DOI:** 10.1155/2022/6243228

**Published:** 2022-08-11

**Authors:** Jonathan Ampah, Komla Agbeko Dzisi, Ahmad Addo, Ato Bart-Plange

**Affiliations:** ^1^Food Technology Research Division, CSIR-Food Research Institute, P. O. Box M20 Accra, Ghana; ^2^Department of Agricultural and Biosystems Engineering, Kwame Nkrumah University of Science and Technology, Kumasi, Ghana

## Abstract

Four mango fruit varieties of average slice thickness 0.6 cm and slice area 10 cm^2^ were dried using a mechanical dryer at varied temperatures, 55°C, 65°C, and 75°C. In general, the moisture content (MC) for all samples analyzed decreased with increasing drying time. Palmer and Haden varieties recorded the lowest MCs of 8.7% (w.b.) and 9.3% (w.b.), respectively, when dried for 14 h at 65°C. Palmer variety with the highest initial MC of 87.2% (w.b.) recorded a low final MC of 8.7% (w.b.) when dried for 14 h at 55°C. Moisture ratio decreased from 1.00 to 0.13, 1.00 to 0.12, 1.00 to 0.12, and 1.00 to 0.10 at 55°C for Kent, Keitt, Haden, and Palmer varieties, respectively. Kent, Keitt, Haden, and Palmer varieties recorded effective moisture diffusivity values of 5.90 × 10^–7^, 6.40 × 10^−7^, 6.57 × 10^−7^, and 7.33 × 10^−7^ m^2^/s, respectively. Vitamin C content of 158.34 mg/100 g recorded for Palmer was highest compared to the other varieties. Activation energy values of samples analyzed were between 19.90 and 25.50 kJ/mol for the drying temperature range. The activation energy recorded by Haden variety was highest compared to the rest. Also, twelve mathematical models were analyzed in predicting the moisture ratio of mango fruit slices during thin layer drying. The results showed that the Midilli, Page, Wang and Singh, and Logarithmic models exhibited supremacy in predicting drying behavior compared to the other eight models.

## 1. Introduction

Mango (*Mangifera indica L*.) is a member of the family Anacardiaceae that also includes important fruit and nut species such as cashew (*Anacardium occidentale*) and Pistachio (*Pistacia vera*) [1]. Currently, Malawi is the largest producer in Africa with 2,083,471 MT followed by Egypt with 1,473,538 MT and Nigeria, 946,695 MT. Ghana, with a production capacity of 112,856 MT, ranks 13^th^ out of the 24 African countries that produce over 10,000 MT of mangoes, mangosteens, and guavas [2]. The Kent fruit is ovate in shape, firm, fleshy, and fibreless with a pleasant taste. The fruits are relatively large and weighs between 500 and 900 g [3]. The Keitt fruit on the other hand is oval in shape, longer, and relatively flatter in length compared to Kent. The Haden fruit is bright yellowish with a deep crimson and oval with a rounded base [4]. Quite on the contrary, the Palmer fruit is yellow-orange with a dark red to crimson blush, oblong with rounded base [4].

Consumption of fruits and vegetables has been found to mitigate problems associated with health, environment, and biodiversity through the reduction of global agricultural greenhouse emissions, reduce deforestation, and prevent various diet-related chronic diseases such as type II diabetes and coronary heart disease [5]. In Ghana, tropical fruits produced in the peak periods are either consumed fresh, sold at relatively cheap prices, or are allowed to go waste due to inadequate processing facilities [6]. Mangoes are highly nutritious and provide a rich source of *β*-carotene, ascorbic acid, pectins, tannins, and minerals like Ca, K, and P [7].

Despite mango being traded worldwide, its high moisture content may be attributed to the high perishable nature exhibited that negatively impacts on trade. Regardless being a rich source of nutrients and vitamin C, its perishable nature limits the scope of utilization. High moisture content of mango makes the fruit susceptible to postharvest losses leading to economic losses during transportation, storage, and processing. This study was necessitated by high postharvest loss recorded in the mango supply chain [8]. A major means of mitigating such losses is through drying. Drying serves as a means of reducing perishability, extending the shelf life, and ensuring regular supply for consumption throughout the year. Drying is necessary to reduce their water activity, prevent microbial spoilage, reduce weight, and decrease packaging, handling, and transportation costs. In general, fruits and vegetables are dried to enhance their storage stability, minimize packaging requirements, and reduce transport weight and consequently transport costs [9]. Lower moisture content prevents the growth and reproduction of microorganisms and causes inactivity of moisture-mediated deteriorative reactions [10, 11]. Dried fruits are a good source of energy because they contain concentrated fruit sugars. Dried foods are high in fiber and carbohydrates and low in fat, making them healthy food choices [12].

Although several researchers have investigated the drying kinetics of various agricultural products such as drying of tomatoes [13] and raw mango slices [14], the drying characteristics of four of the high export-oriented mango varieties in Ghana have not yet been fully elucidated resulting presently in a dearth of information on its behavior during drying. Again, the combined influence of temperature and variety on parameters that impact on food quality and consumer preference such as acidity, color, and vitamin C have not been fully exhausted. Also, several theories and models have been reviewed on the drying mechanism of food materials [13, 15], and capillary and diffusion theories have been found to be most suitable. In the quest to identify an appropriate drying model to describe moisture movement in the fruit, 12 thin layer drying models were evaluated to identify the model(s) of best fit. This study provides an alternative avenue for farmers and processors who may be in a position to venture into the production and sale of dried mango chips locally as well as for export. Mangoes that do not meet fresh export protocols and devoid of the acceptable brix requirement for juice processing may be channeled into dry chips production. Studies into thin layer drying kinetics of mango are important to support the possible boost in commercialization of dried mango chips due to the increased interest in high-quality dried foods with minimum additives and preservatives by consumers. Furthermore, many researchers working on the drying process of agricultural products have been drawn to the importance of preservation of qualitative attributes (such as color and shrinkage) as well as thermodynamic properties linked to reduction in energy consumption [16, 17]. This study assessed the effect of drying temperature and varietal differences on selected moisture properties, pH, acidity, color, and vitamin C of mango. The results from this study may be useful in enhancing drying processes, providing reference for dryer design, and improving physicochemical and nutritional quality of dried fruits.

## 2. Materials and Methods

### 2.1. Sample Preparation and Experimental Procedure

Mangoes were harvested from IdealEbenLiz Farms at Somanya, Yilo Krobo District of the Eastern Region of Ghana. Fruits were stored at 5°C for 24 hours to simulate long distance transport. Drying was carried out at designated temperatures for 8-14 h. Triplicate samples were used in moisture content determination and the averages used for data analysis. When drying was completed, the samples were allowed to cool for 5 minutes and packed into transparent thick polybags. They were then sealed using a heat sealer (Model No. QNS-300 L, Item No. LB032E, 230 V, 50/60 Hz, 450 W, China) and labelled. The samples were stored in a refrigerator at 5°C for further analysis.

### 2.2. Dryer Description

**Figure 1:**
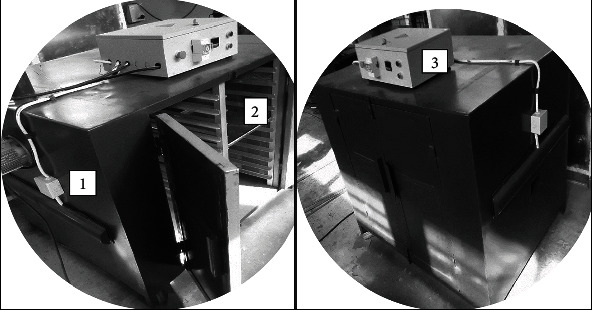
Fabricated mechanical dryer: (1) cabinet body, (2) drying chamber, and (3) control panel.

The mechanical dryer (Figure 1) was constructed based on the design of the R. Royce Industrial Dryer, UK. The major parts of the dryer include the drying chamber which houses the drying trays, the heating elements which serve as the heat source, the control panel which modulates the temperature, and drying time and the exhaust through which evaporated moisture from fruit escapes from the drying chamber. Heating elements (1.5 kW each) were arranged horizontally in a straight line along the midsection of the two internal-facing dryer sides. The current dryer had a capacity of 40 kg with a 20-tray compartment. To achieve thin layer drying characteristics, a 2 kg load per tray design was utilized. All the sides of the cabinet dryer were lagged to minimize heat loss from the dryer to the environment. The drying chamber was divided longitudinally into two halves. The body of the dryer was made from mild steel and coated with antirust paint and food-grade paint. The movable trays, made of aluminium, sat on mild steel angle iron frames with equal spacing to allow for smooth flow of drying air above and beneath the product curtailing the need to stir samples or interchange trays during drying. The dryer was modified to improve drying rate. The initial one (R. Royce model) had both the suction and exhaust at the top (ceiling) section of the cabinet, presenting a complex convection system which sometimes leads to condensation within the dryer. With the current modified version, the suction was placed close to the bottom section of one side, while the exhaust was placed at the top section of the opposite side. This created a simpler convective current path, consequently improving removal of moisture from samples. The control panel was preprogramed to completely shut down the dryer at the designated times.

### 2.3. Moisture Content (MC)

The moisture content of the mango slices was determined using the oven drying method [18]. A Clifton laboratory oven (Model No. NE9-1125, Bennet Scientific Limited, UK) was used in moisture content determination. The initial and final mass (g) of the samples was determined using a precision balance (Mettler Toledo, max load: 5 kg, China). The moisture content wet basis (% w.b.) was determined using

(1) $$\begin{array}{c} {\text{MC}}_{\text{w}.\text{b}.}=\frac{M_{i}-M_{f}}{M_{i}}\times 100\%, \end{array}$$

where *M*_*i*_ is the mass of the mango slice before oven drying (kg) and *M*_*f*_ is the mass of the mango slice after oven drying (kg).

### 2.4. Moisture Ratio (MR)

Moisture ratio is the ratio of the moisture content at any given time to the initial moisture content. Under thin layer drying conditions, the moisture ratio is given as [19]

(2) $$\begin{array}{c} \text{MR}=\frac{M-M_{e}}{M_{o}-M_{e}}\times 100\%, \end{array}$$

where *M* is the average MC at time *t* (kg water/kg dry matter), *M*_*o*_ is the initial MC (kg water/kg dry matter), and *M*_*e*_ is the equilibrium MC.

When analyzed, the *M*_*e*_ values are relatively small compared to *M* or *M*_*o*_ and hence can be considered as negligible. Consequently, MR of the equation was simplified as defined by some researches as follows [13, 20]:

(3) $$\begin{array}{c} \text{MR}=\frac{M}{M_{o}}. \end{array}$$

### 2.5. Drying Rate (DR)

The drying rate was calculated based on weight of water removed per unit time per kilogram of dry matter, expressed in units of kg H_2_O/kg dm/h [19].

The DR is calculated from the following equation:

(4) $$\begin{array}{c} \text{DR}=\frac{M_{t+⧍t}-M_{t}}{⧍t}, \end{array}$$

where *M*_(*t*+∆*t*)_ and *M*_*t*_ are moisture content at time *t* + ∆*t* and *t* (kg water per kg dry solids), respectively, and ∆*t* is time interval (between two consecutive measurements).

### 2.6. Effective Moisture Diffusivity

This describes the diffusion or movement of moisture in food and agricultural products during drying. Other researchers [11] describe moisture diffusivity as a function of material moisture content, material structure, and temperature. Researchers over the years have applied linear regression analysis to fit the experimental data to [13, 15, 21]

(5) $$\begin{array}{c} \text{MR}=\frac{8}{\pi^{2}}\exp\left[\frac{-D_{\text{eff}}}{4l^{2}}\pi^{2}t\right], \end{array}$$

where *D*_eff_ is effective moisture diffusivity (m^2^/s), *t* is the drying time (s), and *l* (m) is half the thickness.

Moisture diffusivity is important in correct simulation of different drying processes [22]. However, moisture diffusivity varies due to numerous factors such as food material properties and process parameters. For a particular material, moisture distribution is influenced by factors like processing temperature, physical structure, moisture content, and porosity [23].

### 2.7. Activation Energy (*E*_*a*_)

A certain minimum amount of energy is required to initiate diffusion of moisture from the food product undergoing drying and that is referred to as the *E*_*a*_. The activation energy is calculated from the effective moisture diffusivity and drying temperature using Arrhenius equation [13, 21].

(6) $$\begin{array}{c} D_{eff=}{Do}_{\exp}\left(-\frac{E_{a}}{R\left(T+273.15\right)}\right), \end{array}$$

where *D*_eff_ is the effective moisture diffusivity (m^2^/s), *D*_0_ is the preexponential factor of Arrhenius equation (m^2^/s), *E*_*a*_ is the activation energy (kJ/mol), *R* is the universal gas constant (kJ/mol K), and *T* is the temperature in Kelvin (K).

### 2.8. Drying Models

During drying, liquid diffusion, vapor diffusion, and capillary forces within the product cause internal mass transfer of water which evaporates upon reaching the product surface [24]. Drying kinetics is influenced by existing drying conditions, type of dryer under use, and characteristics of material to be dried. The drying kinetics models become operative when selecting ideal drying conditions. These conditions are important parameters in assessing equipment design and determining product quality improvement [25, 26].

In describing the drying kinetics, semiempirical equations are best suited when external resistance to heat and mass transfer is negligible [27]. Therefore, semiempirical expressions provide a better choice for analysis where concentration dwells upon engineering design during the drying process. One way of eliminating the impact of external resistance to heat and mass transfer in fruits is to dry in a single thin layer. Accordingly, thin layer drying modelling approach has the potential to estimate drying kinetics from experimental data and improve the drying process. Twelve thin layer drying models were selected for fitting the experimental data as provided in Table 1.

### 2.9. Goodness of Fit

MC data obtained from drying experiment was converted to MR and fitted to 12 thin layer drying models. Nonlinear regression analysis was performed using Minitab Statistical software (version 17.0). The thin layer drying models were compared based on their *R*^2^ (coefficient of determination), *χ*^2^ (MSE) (mean sum of squares of errors), and RMSE (root mean square error). The *R*^2^ is one of main criteria for selecting the best model to describe drying curves [33]. *R*^2^ is equivalent to the ratio of the regression sum of squares (SSR) to the total sum of squares (SST). The *R*^2^ illustrates the proportion of variance accounted for in the dependent variable by the model. It assesses how best the model fits the data and has been used by various authors to evaluate the drying models [13, 19, 33].

The best fit model describing the drying characteristics was chosen based on the highest *R*^2^ value and the lowest *χ*^2^. *χ*^2^ is the mean square of the deviations between the experimental and calculated moisture levels [33].

(7) $$\begin{array}{c} x^{2}=\frac{\sum_{i=1}^{N}{\left({\text{MR}}_{\text{pre},i}-{\text{MR}}_{\exp,i}\right)}^{2}}{N-z}, \end{array}$$

(8) $$\begin{array}{c} \text{RMSE}=\sqrt{\frac{1}{N}}\overset{N}{\underset{i=1}{\sum }}{\left({\text{MR}}_{\text{pre},i}-{\text{MR}}_{\exp,i}\right)}^{2}, \end{array}$$

where MR_expt,*i*_ and MR_pre,*i*_ are the experimental and predicted dimensionless MR, respectively, *i* is the experimental observation in a given *N* total number of observations, and *z* is the number of constants in the model.

The RMSE represents the noise in the data [34] and is given as shown in Equation (8).

### 2.10. pH, Acidity, Color, and Vitamin C of Mango

**Figure 2:**
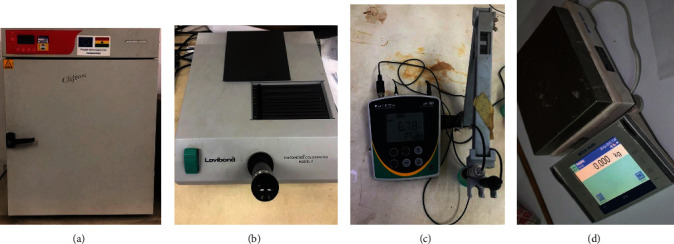
Applied equipment: (a) Clifton laboratory oven, (b) Lovibond tintometer colorimeter, (c) pH-700 meter, and (d) precision balance.

The pH of fruit slices was determined using approved methods of the Association of Official Analytical Chemists. The Eutech pH-700 meter (ECFC7252101B-electrode, Thermo Fisher Scientific, China) was used in pH determination. Acidity was determined using standard laboratory methods of analysis which involve measuring 10 ml of sample into a 250 ml conical flask, adding 100 ml distilled water and 3 drops of 1% phenolphthalein indicator, and finally titrating against 0.1% N sodium hydroxide until the solution turned pink. Color values were attained by placing small pieces of mango slices unto the illuminated slide of the Lovibond Tintometer Colorimeter (Model F, UK) while adhering to standard procedures. Vitamin C values were obtained through standard titration procedures [35].  Figure 2 displays some of the equipment that were used in data collection and analyses.

### 2.11. Statistical Analysis

Data analysis and plotting of graphs were carried out with Microsoft Excel 2016 (NY, USA) and Stata V.12 (TX, USA). The empirical model constants for the drying models were determined using nonlinear regression. The experimental data for pH, acidity, color, and vitamin C were subjected to ANOVA using Stata Version 12 (Stata Corp., TX, USA). The results were expressed as means ± standard deviation, and separation of means was performed by the Bonferroni adjustment at 5% significance level.

## 3. Results and Discussion

### 3.1. Moisture Content

**Figure 3:**
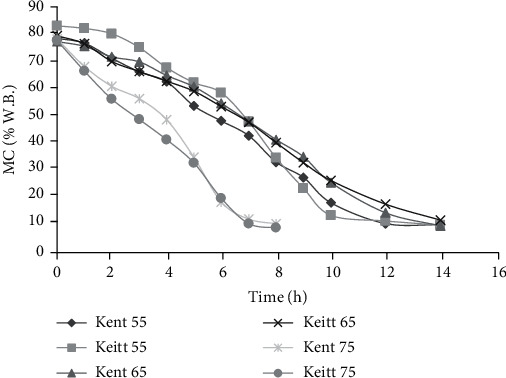
Moisture content of Kent and Keitt during drying.

Figure 3 shows the relationship between MC and time at different drying temperatures. It can be observed that as drying time increased, samples MC decreased [12, 36]. The initial moisture content recorded for Kent, Keitt, Haden, and Palmer mangoes was in the range 73.21-78.47%, 76.53-83.07%, 81.55-84.60%, and 82.8-87.20%, respectively. Also, the final moisture content of Kent, Keitt, Haden, and Palmer mangoes was found to be in the range of 6.72-10.61%, 5.60-11.87%, 8.50-11.20%, and 7.50-11.10%, respectively. The moisture content results of both wet and dried samples were found to be similar to that of mango [29] and Cardaba banana [21]. Generally, the Palmer and Haden varieties recorded higher initial MC in excess of as much as 10% above the Kent and Keitt varieties. Variations observed in moisture content, drying rate, and diffusivities for mango samples analyzed in the present study may be related to varietal and growth conditions of Palmer and Haden [37]. Drying at 55°C employed the longest time, while that of 75°C recorded the shortest time of 8 h. This is corroborated by Hashjin et al. [38] for drying food samples at 50°C, 60°C, and 70°C.

Both Palmer and Haden varieties displayed gradual decrease in MC until from the 2^nd^ hour at 75°C and the 6^th^ hour for samples dried at 55°C and 65°C. The flat nature of the graph towards the end of drying is symbolic of very little moisture loss from mango slices towards the end of the drying process. As drying occurs and MC decreases, the dry matter content of sample increases relative to water content [38] causing a reduction in the rate of moisture removal from the sample. The lengthy drying time of samples at 55°C may be related to the inability of surface moisture to quickly evaporate due to the low drying temperature which poorly influences internal capillary forces.

The presence of little fiber in palmer [4] may be responsible for the quick loss of moisture from the surface as well as within the fruit due to fast evapo-capillary movement of water. This increased the drying rate and gave Palmer variety its characteristic steep slope compared to the other varieties. Palmer variety recorded the least change in MC during the final 4 hours of drying at 55°C. This may be due to the slow removal of internally bound water after all free surface water has been evaporated.

### 3.2. Moisture Ratio

**Figure 4:**
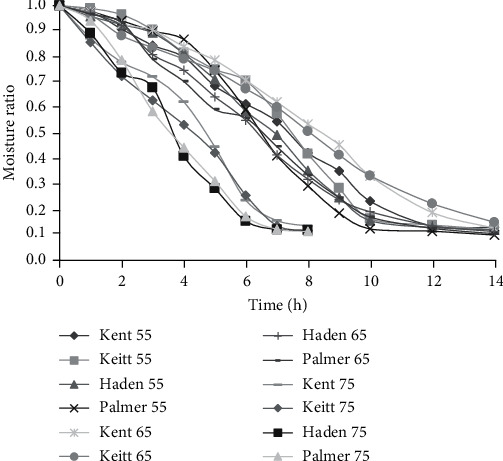
Moisture ratio curves at 55°C, 65°C, and 75°C.

The ratio of free water not yet removed to the total free water that was initially available is the MR [39]. The MR graph as depicted in Figure 4 followed by a similar path to that of MC.

The lowest MR of 0.0998 and 0.1048 was recorded by the Palmer variety dried at 55°C and 65°C, respectively. This is likely due to the high MC that was initially present compared to the low MC recorded at the end of drying, implying a wide difference between the initial and final MCs. Among the four varieties, the highest MR at the end of the drying process was recorded by Keitt dried at 65°C followed by Kent dried at 75°C. Research carried out on mango [40] at varied temperatures (60°C, 70°C, and 80°C) recorded final MR comparable to values obtained in the current study. Similar observations of decreasing MR with increasing drying time have been recorded in carrot pomace [22], roselle [19], and mango [13].

### 3.3. Drying Rate

Drying at 55°C showed high peak points between the 4^th^ and 8^th^ hours for all four varieties. During drying at 65°C, Kent and Keitt varieties exhibited very slow rise from onset of drying up till the 10^th^ hour, after which a rise was recorded moving towards the 12^th^ hour. Haden recorded a high peak of 22.35 during drying at 75°C.

It was noted that there was no constant rate drying period; drying took place predominantly in the falling rate period indicating that diffusion was the main phenomenon by which drying occurred. Similar results regarding the diffusion mechanism being the dominant controlling mechanism of the drying process have been reported extensively in literature such as the drying of mango [29] and date palm [39].

### 3.4. Effective Moisture Diffusivity

**Figure 5:**
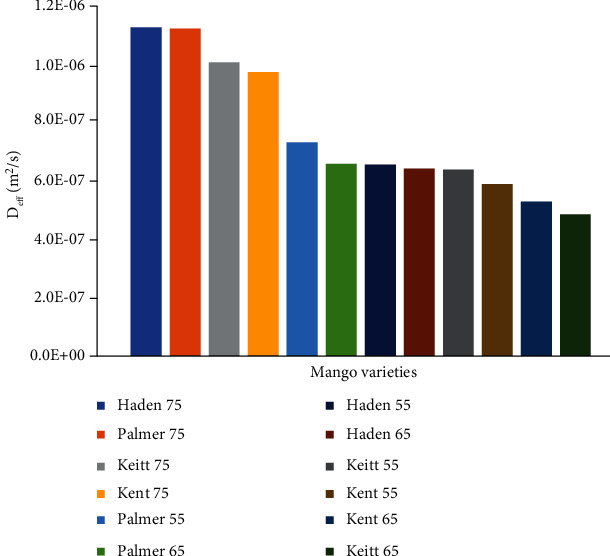
Effective moisture diffusivity of mango.

Figure 5 displays the effective moisture diffusivities of the four mango varieties at 55°C, 65°C, and 75°C.

Haden and Palmer recorded the highest diffusivities of 1.1274 × 10^−6^ and 1.1231 × 10^−6^, respectively, at 75°C. The high diffusivities of these 2 varieties compared to Kent and Keitt may be attributed to the availability of larger quantities of free water present in the fruit and the varied material structure of the four varieties [12, 37]. *D*_eff_ recorded in this study was similar to studies carried out by Corzo et al. [41] between 50°C and 80°C on Hilacha mango slices. Diffusivity values were found to increase with increasing drying temperatures. From this study, it may be concluded from the definition of *D*_eff_ that the movement of moisture in mango slices occurred by diffusion in the falling rate period and is influenced to an extent by the drying temperature [39, 42]. The values obtained in this study were similar to that for Cardaba banana (1.46 × 10^−8^ at 50°C to 1.45 × 10^−6^ at 70°C) [21].

### 3.5. Activation Energy

The highest *E*_*a*_ of 25.40 kJ/mol was recorded for the Haden variety and the lowest of 19.95 kJ/mol for Palmer. From this, it can be deduced that among the four varieties, Palmer required the least amount of energy to initiate diffusion of moisture during drying. Activation energies were found to be similar to that recorded for apple (19.96 kJ/mol) [43]. Conversely, *E*_*a*_ values were lower than that recorded for banana (51.21 kJ/mol) [42] and red chilli (41.95 kJ/mol) [44]. Differences in *E*_*a*_ may be ascribed to differences in chemical composition and cellular structure of different agricultural products [37].

### 3.6. Thin Layer Drying Models

Table 2 summarizes the outcome of the nonlinear regression modelling comparing their *R*^2^, *χ*^2^, and RMSE. These models were selected due to their wide application to varied food and agricultural materials. MC data obtained from drying experiment was converted to MR and fitted to 12 thin layer drying models. Nonlinear regression analysis was performed using Minitab Statistical software (version 17.0).

The lowest *R*^2^ value was recorded by the Wang and Singh model (Kent 75°C). Out of the 12 drying processes, 5 of them were best described by the Midilli model, 2 by Page, 2 by Modified Page I, 2 by Logarithmic, and 1 by Wang and Singh models. Hence, the Midilli models may be assumed to be the predominant model that best describes the thin layer drying behavior of mango slices [14]. It came to light that at lower temperature of 55°C, the Page and Midilli drying models were the best descriptors. The drying kinetics of Kent and Keitt samples dried at 75°C were best described by Logarithmic and Wang and Singh models.

It was determined that the value of the drying rate constant (*k*) increased with an increase in temperature with reference to the Midilli model which was the dominant descriptive model. This implies that with increase in temperature, drying curve becomes steeper indicating increase in drying rate. The Henderson and Pabis and Modified Henderson and Pabis models were found to be poor descriptors of drying in fruits and vegetables with the exception of apple and pumpkin [25]. The presence of 6 terms in the equation makes it a complex one that requires more than 6 datapoints to compute the outcome effectively.

**Figure 6:**
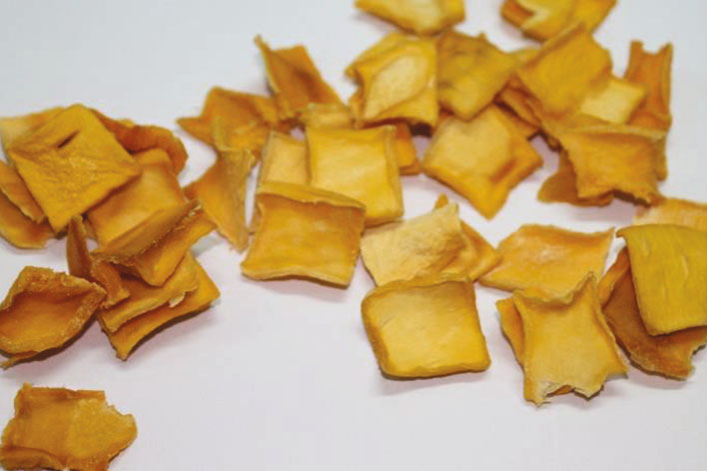
Dried mango sample.

By analyzing broadly twelve thin layer drying models, it was documented that only five of them supported the thin layer kinetic modelling of the four mango varieties used in the current study. Previous studies concentrated on mostly the Newton and Page models [28, 38], but the importance of other models such as the Logarithmic and Wang and Singh models have been highlighted in this study. Most research on kinetics studies utilized lab-scale dryers in its determination [10, 12]. However, due to the strong motivation of providing alternative sources of income for farmers and processors, a modified scaled-down version of an industrial dryer was incorporated in this research serving as a better reference for industrial operations.  Figure 6 shows dried samples of Keitt mango.

### 3.7. Predicted MR vs. Experimental MR

**Figure 7:**
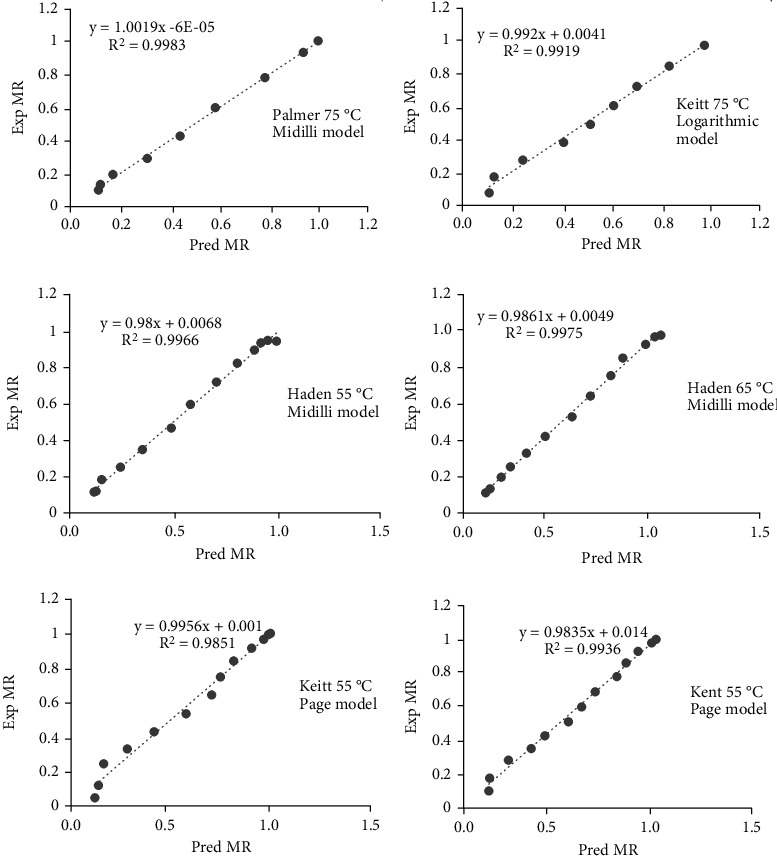
Model fitting for Kent, Keitt, Haden, and Palmer varieties at 55°C, 65°C, and 75°C.

The accuracy of the established model for the thin layer drying process was evaluated by comparing the predicted MR with observed MR (Figure 7). The models showed very good fit between the experimental and predicted MR, which confirms their suitability in describing the mechanical drying of Kent, Keitt, Haden, and Palmer varieties at the three temperature points. Predicted data generally banded around the straight line which showed the suitability of the logarithmic model for Keitt variety dried at 75°C, Page model for Kent variety at 55°C, Midilli model for both Palmer at 75°C, and Haden at 65°C in describing the drying behavior of mango slices. In other research, the Page model gave the highest correlation between the experimental and predicted moisture MR [29]. The Midilli model was found to provide the best correlation of mango slices dried using the mechanical dryer [40].

### 3.8. pH, Acidity, Color, and Vitamin C of Mango

From the results of statistical analysis displayed in Table 3, it can be deduced that varietal differences did not have significant effect on initial acidity of food samples but rather on the final acidity. Significant differences were recorded between Keitt and Haden varieties. Kent and Palmer were however found to exhibit statistically similar acidity characteristics but slightly different from Keitt and Haden upon completion of drying. Analysis of the initial and final acidity values for all four mango varieties were found to be within the range of 3.48–6.40 g/100 g, which is relatively lower than what was reported by Tasie et al. [17]. A study by Mwamba et al. [45] also reported an acidity of 0.86 g/100 g in fresh mangoes and 1.65–2.65 g/100 g in dried mangoes which is slightly lower than the data recorded in the current research. Again, the results from all four varieties indicate an increase in acidity with no significant difference between initial values. Increase in acidity of dried samples indicates the conversion of some carbohydrates to acids [46] and the occurrence of a level of fermentation [45, 47]. Dereje and Abera [48] in their study on fresh mangoes reported average acidity value of 1.92 g/100 g. This is relatively higher than the values of the current study. The acidity of Tommy Atkin variety was determined to be 0.88 g/100 g and similar to that documented for Keitt in this study. The differences in fresh mango acidity observed in this study and the referenced values could be due to the varieties of mangoes used and the drying method applied. High acidity values are an indication of sourness. Hence, among the four varieties, Palmer may taste sourest in the dried form compared to Haden which may taste sweetest.

pH observed in this study was lower than that reported by Tasie et al. [17] in their study of different mango varieties (ranging from 3.86 to 4.73). Mwamba et al. [45] also reported a higher pH value (3.71) in fresh mangoes than what was observed in this study. pH of dried samples was slightly higher than fresh samples. This may be linked to the concentration of organic acids owing to the reduced moisture content during drying [48]. Significant differences existed between the final pH values of all varieties with the exception of the pairings: Kent and Keitt Haden and Palmer. Averagely, Kent and Palmer registered the highest and lowest pH values, respectively.

Color which is an important parameter with reference to marketability was also investigated. It came to light that drying at lower temperature of 55°C provided the highest intensities for colors *R* and *Y*. Drying at 75°C registered the lowest color values. For the processor and quality manager who wish to appeal to customers, it is worth noting that drying beyond 65°C diminishes the attractiveness of mango chips. Haden recorded the highest *R* and *Y* values of 2.69 and 7.87, respectively, after drying. Keitt documented the lowest values for both colors after drying. It may be assumed that Haden registered the brightest colors and as such would be preferable by consumers due to its attractiveness. There were significant differences between Kent and Palmer varieties considering both *R* and *Y* values. The Haden variety maintained its yellowish color throughout the drying process. *Y* intensity diminished for the other three varieties (Kent, Keitt, and Palmer) from onset to end of drying. It can be inferred that variety acting independently had a significant effect on *R* and *Y* values, particularly on Haden variety. For Kent variety, the lowest *R* value (1.0) was recorded in samples dried at 65°C, followed by those dried at 75°C. The highest *R* value was recorded by samples dried at 55°C from the 7^th^ to 10^th^ hours, followed by those dried at 55°C during the 6^th^ hour. The highest *Y* value (20.0) was recorded by samples dried at 55°C. *Y* values for Keitt samples hovered around 10.0 for major part of the initial drying hours and reduced to less than 5.0 during the latter part. Samples dried at 75°C recorded the least *Y* (2.4), and those dried at 55°C recorded the least *R* (0.5). Haden samples dried at 65°C recorded the highest *Y* value (9.05) at the end of the drying process. For Palmer, *R* values showed very little variation among the different drying regimes. The highest average final *Y* value (7.75) was recorded in samples dried at 65°C. Appearance and color have been identified as important parameters in food selection as such food products with uncharacteristic color changes may be rejected [49].

Again, this study did not only stick with drying kinetic modelling of mango but went further to determine the impact of temperature and variety on some chemical and nutritional properties. For example, with respect to vitamin C, Palmer was found to exhibit high values at the end of the drying period, and this was followed by Keitt. Such information may be valuable to nutritionists in selecting mango varieties with high nutritional value when targeting vitamin C-deficient children and seniors. The average initial vitamin C content of the varieties analyzed oscillated from 17.20 to 40.83 mg/100 g. Research conducted on vitamin C content of Tommy Atkins, Sabre, Zill, Peach, Rosa, and Phiva [50] found results similar (17.01 to 50.71 mg/100 g) to the current study. Research carried out by Sogi et al. [51] on Tommy Atkin mangoes recorded ascorbic acid content of the fresh-cut mango cubes to be 15.97 mg/100 g FW which is slightly lower compared to values obtained in this study. Another study reported vitamin C content of fresh mangoes to be 34.67 mg/100 g [52], which was higher than that recorded for Kent, Keitt, and Haden varieties in this study. Initial values did not record any significant differences. The average final vitamin C contents however displayed the presence of a significant difference in the Palmer variety. Although Kent, Keitt, and Haden varieties were not statistically different, their mean values exhibited some variations. Generally, the Palmer variety recorded the highest vitamin C values for final dried product.

## 4. Conclusion

Drying time reduced considerably from 14 h at 55°C to 8 h at 75°C, a difference of 6 h culminating in 57% time savings. Cost savings upwards of GH10.00/h was realized when samples were dried at 75°C. Increase in drying temperature increased drying rates and ultimately decreased drying time. High drying rates were recorded among all four mango varieties between the 4^th^ and 8^th^ hours of drying with Keitt and Palmer recording in excess of 10 (kg water/kg dry matter/h) over Kent and Haden during this period. Overall, Haden and Palmer varieties recorded the highest initial MC as well as the lowest final MC. The largest moisture diffusivities were recorded during drying at 75°C. This is probably due to the relatively high drying temperature which causes the fast movement of large amounts of moisture within a shorter time. The Midilli model displayed the best fit (*R*^2^, 0.998236; RMSE, 0.03820492; *χ*^2^, 0.0004071) as well as the highest *R*^2^ values among all the temperature-variety combinations researched. Hence, the Midilli model was found to be the most accurate predictor of the general drying behavior of mango, more specifically Haden and Palmer varieties. The Page and Modified Page models predominantly were better descriptors for the Keitt and Kent varieties. The models identified will be useful in determining drying duration, moisture ratio, and generally for purposes of optimization and simulating the drying process in mango. Keitt and Kent varieties dried at 55°C and 65°C recorded the least diffusivities which suggests that a minimum amount of energy is required to initiate diffusion of moisture from these two. The Haden variety, by contrast, recorded the highest activation energy implying that it required the largest amount of energy to initiate diffusion of moisture during drying. Generally, the locally developed mechanical dryer is effective in drying mango slices to storable MC with the added advantage of safe production due to noncontact by pests, insect, and dust and shorter drying times at higher temperatures. It is also worth noting that due to the difficulty and expenses involved in conducting full-scale drying tests, the study of drying kinetics is applied to model the moisture removal process in relation to other process variables and provide basis for scale-up or control of operations [53]. The study of drying kinetics is important for the design of dryers and has cost and quality implications for dried foods [54]. From the study, drying Kent and Keitt varieties beyond 8 h at 75°C is unnecessary and a waste of time and energy with negative cost elements involved. This was most visible where the storable MC was achieved at 8 h. Hence, drying beyond this point would have the potential to reduce the size of food samples while destroying heat-sensitive nutrients.

Acidity increased with increasing time for all varieties. Final pH values ranged between 3.25 and 3.77, with Kent recording the highest. With the exception of the Haden variety which registered averagely higher *R* and *Y* values, there were no significant differences between final color values of the other three varieties. From the research, drying at lower temperatures realized high intensities of colors *R* and *Y* that can enhance marketability compared to drying at higher temperatures such as 75°C.

Vitamin C increased drastically between the fresh and dried samples most likely due to loss in moisture and subsequent concentration of nutrients. It may be concluded that varietal effect on the vitamin C content of mango had no significant effect on initial and final values with the exception of the Palmer variety which is in contrast to research carried out on tomato [55]. The high vitamin C content of dried Palmer samples obtained presents an opportunity for processors and nutritionists to recommend these dried chips to nutrient-deficient children and invalids to boost their immune system.

It was observed that vitamin C content was dependent on both conditions of cultivation and variety. Further research additionally attributed the vitamin C differences to level of ripeness, growth stage, geographical distribution, and the nature of postharvest handling practices, implying that variety may not be solely responsible for vitamin C content in mango but rather a complex relation of other physiological and agricultural factors.

## 5. Future Outlook

- Further research into the use of alternative drying methods such as microwave, osmotic, and hybrid systems may be explored to curtail total dependency on cabinet drying which maintains high electricity consumption
- Explore the production of vitamin C effervescent tablets from dried mango chips
- Mango is rich in *β*-carotene, which has been researched to be associated with reduced risk of cancer. Hence, further studies should look into the effect of drying temperatures on *β*-carotene content in mango
- Additionally, research into the identification of novel products that may be derived from dried mango chips such as incorporation into yoghurts and meals for toddlers and the aged should be explored further

## Figures and Tables

**Table 1:** Mathematical models for describing thin layer drying characteristics.

Model name	Model equation	References
Newton	MR = exp (−*kt*)	[28]
Page	MR = exp (−*kt*^*n*^)	[29]
Modified Page I	MR = exp[−(*kt*)^*n*^]	[14]
Modified Page II	MR = *k* exp (−*t*/*d*^2^)*^n^*	[30, 31]
Henderson and Pabis	MR = *a* exp (−*kt*)	[19]
Modified Henderson and Pabis	MR = *a*exp(−*kt*) + *b*exp(−*gt*) + *c* exp (−*ht*)	[31]
Logarithmic	MR = *a* exp (−*kt*) + *c*	[30]
Wang and Singh	*MR* = 1 + *at* + *bt*^2^	[20]
Peleg	MR = 1 − *t*/(*a* + *bt*)	[32]
Midilli	MR = *a* exp (−*kt*^*n*^) + *bt*	[27]
Verma et al.	MR = *a* exp (−*kt*) + (1 − *a*)exp(−*gt*)	[31]
Diffusion approach	MR = *a* exp (−*kt*) + (1 − *a*)exp(−*kbt*)	[30]

Where *a*, *b*, *c*, *g*, *h*, and *n* are empirical constants, *k* drying constant, *t* drying time, and MR moisture ratio.

**Table 2:** Mathematical modelling for thin layer drying of mango.

Product	Model of best fit	Model constants					*R* ^2^	RMSE	*χ* ^2^
		*k*	*n*	*a*	*b*	*c*			
Keitt 55°C	Page	0.00754	2.27039				0.982037	0.045460	0.0026303
Kent 55°C	Page	0.02189	1.75785				0.990438	0.034527	0.0011921
Haden 55°C	Midilli	0.00391	2.75463	0.94109	0.00799		0.996083	0.020319	0.0002372
Palmer 55°C	Midilli	0.00154	3.29568	0.95181	0.00798		0.997250	0.018354	0.0002572
Keitt 65°C	Logarithmic	-0.01933		-2.87563		3.88808	0.990748	0.025280	0.0008308
Kent 65°C	Modified Page I	0.09879	2.38954				0.993829	0.020179	0.0005172
Haden 65°C	Midilli	0.01603	2.10375	0.96611	0.0068		0.997278	0.016432	0.0002952
Palmer 65°C	Midilli	0.02697	1.81864	0.98768	0.00371		0.994093	0.024064	0.0009220
Keitt 75°C	Logarithmic	0.03494		3.73859		-2.74532	0.991918	0.026555	0.0010578
Kent 75°C	Wang and Singh			-0.103255	-0.001574		0.976078	0.046908	0.0028290
Haden 75°C	Modified Page I	0.22459	1.66225				0.986102	0.038390	0.0022107
Palmer 75°C	Midilli	0.07838	1.77867	1.00744	0.00726		0.998236	0.038205	0.0004071

**Table 3:** Chemical and nutritional properties of four mango varieties.

Variety	Acidity g/100 g (I)	Acidity g/100 g (F)	Vit C mg/100 g (I)	Vit C mg/100 g (F)	pH (I)	pH (F)
Kent	0.94 ± 0.40^a^	2.97 ± 0.80^ab^	17.20 ± 7.48^a^	66.41 ± 20.15^b^	3.77 ± 0.31^a^	3.95 ± 0.23^a^
Keitt	0.85 ± 0.28^a^	3.85 ± 1.16^a^	28.91 ± 21.11^a^	98.70 ± 38.67^b^	3.54 ± 0.36^ab^	3.85 ± 0.26^a^
Haden	0.62 ± 0.52^a^	2.32 ± 1.76^b^	31.52 ± 35.43^a^	83.18 ± 34.45^b^	3.36 ± 0.23^bc^	3.54 ± 0.31^b^
Palmer	0.66 ± 0.44^a^	3.16 ± 2.10^ab^	40.83 ± 41.41^a^	158.34 ± 92^a^	3.25 ± 0.17^c^	3.32 ± 0.17^b^

Means in the same column with the same superscript letters are not significantly different (*p* > 0.05). “I” and “F” represent initial (fresh) and final (dried) values.

## Data Availability

All data are included in the manuscript.

## Nomenclature

MT: Metric ton
kg: Kilograms
g: Grams
h: Hours
°C: Degree Celsius
W: Watts
kW: Kilowatts
kJ: Kilojoule
t: Drying time
m: Meter
w.b: Wet basis
MC: Moisture content
MR: Moisture ratio
DR: Drying rate
D_eff_”: Effective moisture diffusivity
E_a_: Activation energy
*R* ^2^: Coefficient of determination
RMSE: Root mean square error
R: Mean color red value
Y: Mean color yellow value.
